# Supplementary material for: Neuroblastoma arises in early fetal development and its evolutionary duration predicts outcome
Source: Nat Genet. 2023 Mar 27;55(4):619–30. doi: 10.1038/s41588-023-01332-y (PMC10101850; doi:10.1038/s41588-023-01332-y)
Supplement: Supplementary file 1 — Supplementary notes 1 and 2 and tables 11 and 12. [file 41588_2023_1332_MOESM1_ESM.pdf]

nature genetics

Article

https://doi.org/10.1038/s41588-023-01332-y

Neuroblastoma arises in early fetal development and its evolutionary duration predicts outcome

In the format provided by the authors and unedited

nature genetics

Article

https://doi.org/10.1038/s41588-023-01332-y

Neuroblastoma arises in early fetal development and its evolutionary duration predicts outcome

In the format provided by the authors and unedited

Supplementary Table 11. Prior probabilities to model neuroblastoma initiation.

Parameter	Density distribution
N	Log-uniform for $10^3 \leq N \leq 10^9 f$
μ	Uniform for $0 \leq \mu \leq 15$
δ_1/λ_1	Uniform for $0 \leq \delta_1/\lambda_1 \leq 0.9$
δ_2/λ_2	Uniform for $1.01 \leq \delta_2/\lambda_2 \leq 1.5(\delta_2/\lambda_2)$
μ_1	Log-uniform for $10^{-9} \leq \mu_1 \leq 10^{-4}$
μ_2	Log-uniform for $10^{-9} \leq \mu_1 \leq 10^{-4}$
r	Uniform for $0 \leq 1/r \leq 0.999$
ν_2	Uniform for &0.01 $\leq \nu_2 \leq 0.5$

Supplementary Table 12. Prior probabilities to model neuroblastoma progression.

Parameter	Density distribution
n_{clonal}	Uniform for $0 \leq n_{\mathrm{clonal}} \leq 10^4$
μ	Uniform for $0 \leq \mu \leq 20$
δ_T/λ_T	Uniform for $0 \leq \mu \leq 0.99$

Supplementary Note 1. Modeling neuroblastoma initiation in an initially expanding and then contracting population.

(a) Derivation of equations (14a-c). Let P_t be the probability of experiencing two oncogenic events in neural precursors that initially expand and then decay. Consider three cases of how to distribute the two events over the two phases of precursor dynamics: (I) both oncogenic events occur during precursor expansion, associated with probability $P_{\mathrm{MRCA,I}}$; (II) the first oncogenic event occurs during precursor expansion, the second during precursor contraction, associated with probability $P_{\mathrm{MRCA,II}}$; (III) both oncogenic events occur during precursor contraction, associated with probability $P_{\mathrm{MRCA,III}}$. To model Case I, we adopted results from Iwasa et al.[1] and Haeno et al.[2] on the acquisition of two mutations in an exponentially growing population. Briefly, the number of normal cells is deterministically modeled, while cells with the first event are subject to stochastic fluctuations according to a linear birth-death process. We here simplified the setup slightly by assuming that the first mutant is neutral during expansion because of the overall high survival probability during this phase.

At each time point, M_1 cells are generated according to equation (8a) and survive with the non-extinction probability $\nu_1 = 1 - \frac{\delta_1}{\lambda_1}$. An M_1 cell experiences the second oncogenic event with probability μ_2 during each cell division and subsequently survives with probability $\nu_{2,\mathrm{E}} = 1 - \frac{\delta_1}{s\,\lambda_1}$, equation (12a). The probability of at least one type-2 cell at time t is given by[2]

$$P_{\mathrm{MRCA,I}} = \sum_{x=1}^{N(t)-1} e^{-\mu_1(x-1)}\left(1 - e^{-\mu_1}\right)\left(1 - \exp\left\{-\frac{\mu_2 M_1(t) F}{1-\delta_1/\lambda_1}\right\}\right), \tag{S1}$$

where $F = \int_0^1 \nu_{2,I} / \left(\nu_{2,I} z^{\alpha}\right) dz$ and $\alpha = \frac{\delta_1 - s\lambda_1}{\delta_1 - \lambda_1}$. This is equation (14a) in Methods.

For Case II, the first oncogenic event occurring during neuroblast expansion and the second event occurring during decay, we approximated M_1 deterministically, using the time-dependent mean of cells with the first event, and then described acquisition of the second event stochastically. As before, we assumed that new M_1 cells are produced at rate $\mu_1 \lambda_1 N(t) dt$ during tissue expansion [equation (10)], and divide and die at rates λ_1 and δ_1, respectively. Thus the

average number of M_1 cells at T is given by

$$M_1(T) = \int_0^T \mu_1\lambda_1 N(t')e^{(\lambda_1-\delta_1)(T-t')}dt' = \mu_1\lambda_1 T N(T).$$

During contraction the fitness advantage of M_1 cells becomes relevant, which we choose to as a reduction of cell loss proportional to $\frac{1}{r}, r > 1$. Thus, during precursor differentiation, the number of cells with a first hit acquired during expansion, $M_{1,\mathrm{II}}(t)$, evolves according to

$$M_{1,\mathrm{II}}(t) = M_1(T)e^{(\lambda_2-\delta_2/r)(t-T)}; t \geq T.$$

The probability that a surviving cell from this population experiences a second oncogenic event before t, $P_{\mathrm{MRCA,II}}$, can be computed by first determining $\overline{P_{\mathrm{MRCA,II}}} = 1 - P_{\mathrm{MRCA,II}}$, the probability that the second event has not happened until t. Then, the probability of not experiencing a successful second event within a short time span Δt is given by:

$$\overline{P_{\mathrm{MRCA,II}}(t+\Delta t)} = \overline{P_{\mathrm{MRCA,II}}(t)}\big(1 - \mu_2\lambda_2\nu_{2,\mathrm{D}}M_{1,\mathrm{II}}(t)\Delta t\big);\ t \geq T,$$

where $\nu_{2,\mathrm{D}} = 1 - \frac{\delta_2}{s\lambda_2}$ is the survival probability of the second mutant during decay. In the limit $\Delta t \to 0$ we obtain the differential equation

$$\frac{d\overline{P_{\mathrm{MRCA,II}}}}{dt} = -\mu_2\lambda_2\nu_{2,\mathrm{D}}M_{1,\mathrm{II}}(t)\overline{P_{\mathrm{MRCA,II}}};\ t \geq T,$$

which is solved by

$$\overline{P_{\mathrm{MRCA,II}}} = \exp\left(-\frac{\mu_1\mu_2\lambda_1\lambda_2\nu_{2,\mathrm{D}}T}{\lambda_2-\delta_2/r}N(T)\left\{e^{(\lambda_2-\delta_2/r)(t-T)} - 1\right\}\right);\ t \geq T,$$

yielding equation (14b):

$$P_{\text{MRCA,II}} = 1 - \exp\left(- \frac{\mu_1 \mu_2 \lambda_1 \lambda_2 \nu_{2,\text{D}} T}{\lambda_2 - \frac{\delta_2}{r}} N(T) \left\{ e^{\left(\lambda_2 - \frac{\delta_2}{r}\right)(t-T)} - 1 \right\} \right);\ t \geq T.$$

Similar to Case II, we modeled Case III (both oncogenic events taking place during contraction) using a deterministic approximation for the number of cells with the first event. During contraction, new M_1 cells are produced at rate $\mu_1 \lambda_2 N(t)$ and divide and die at rates λ_2 and δ_2/r, respectively. The number of M_1 cells that were born after T, $M_{1,\text{III}}$, is thus given by

$$M_{1,\text{III}}(t) = \int_T^t \mu_1 \lambda_2 N(t') e^{(\lambda_2 - \delta_2/r)(t-t')} dt'$$

$$= \frac{\mu_1 \lambda_2}{\delta_2(1/r - 1)} \frac{N(T) e^{(\lambda_2 - \delta_2/r)t}}{e^{(\lambda_2 - \delta_2)T}} \left(e^{\delta_2(1/r-1)t} - e^{\delta_2(1/r-1)T)}\right); t \geq T.$$

Again, we computed the probability of the second oncogenic event occurring before t, $P_{\text{MRCA,III}}$, by determining $\overline{P_{\text{MRCA,III}}} = 1 - P_{\text{MRCA,III}}$ from

$$\overline{P_{\text{MRCA,III}}(t + \Delta t)} = \overline{P_{\text{MRCA,III}}(t)}\left(1 - \mu_2 \lambda_2 \nu_{2,\text{D}} M_{1,\text{III}}(t) \Delta t\right); t \geq T.$$

Taking the limit $\Delta t \to 0$ and solving the differential equation

$$\frac{d\overline{P_{\text{MRCA,III}}}}{dt} = -\mu_2 \lambda_2 \nu_{2,\text{D}} M_{1,\text{III}}(t) \overline{P_{\text{MRCA,III}}}; t \geq T,$$

we arrive at equation (14c):

$$P_{\text{MRCA,III}} = 1 - \exp\left(- \frac{\mu_1 \mu_2 \lambda_2^2 \nu_{2,\text{D}} N(T)}{\delta_2 \left(\frac{1}{r} - 1\right)} \left\{ \frac{e^{(\lambda_2 - \delta_2)(t-T)} - 1}{\lambda_2 - \delta_2} - \frac{e^{\left(\lambda_2 - \frac{\delta_2}{r}\right)(t-T)} - 1}{\lambda_2 - \frac{\delta_2}{r}} \right\} \right); t \geq T.$$

(b) Derivation of equations (16a,b). In order to solve equation (15), the probability that the first oncogenic event happened before t_1 given the second event at t_2, we again distinguished the

three cases as above. In Case I (both oncogenic events take place during precursor expansion) $t_1 \leq T$ and $t_2 \leq T$. At t_1 the expected number of newly born M_1 cells is $= \mu_1\lambda_1 N(t_1)$. These cells expand at rate $\lambda_1 - \delta_1$ until t_2. We thus have

$$M_1(t_2|t_1; t_2 < T) = \mu_1\lambda_1 N(t_1)N(t_2 - t_1) = \mu_1\lambda_1 N(t_2)$$

M_1 cells at t_2 descending from an ancestor that acquired the first oncogenic event at t_1. Note that $M_1(t_2|t_1; t_2 < T)$ is independent of t_1. Thus, if both events occurred during exponential expansion, every $t_1 \leq t_2$ has equal probability of being the time point of ECA emergence. The probability that the ECA dates back to latest t_1, given that the MRCA dates back to t_2, is accordingly given by

$$P(t_1|t_2; t_2 \leq T) = \frac{t_1}{t_2},$$

which is the first of equations (16). In Case II (the first oncogenic event occurs during precursor expansion, the second during precursor contraction), $t_1 \leq T$ and $t_2 > T$. At $t_2 > T$ we expect

$$M_1(t_2|t_1; t_1 \leq T, t_2 > T) = M_1(T|t_1; t_1 \leq T)e^{\left(\lambda_2 - \frac{\delta_2}{r}\right)(t_2 - T)} = \mu_1\lambda_1 N(T)e^{(\lambda_2 - \delta_2/r)(t_2 - T)} \tag{S2}$$

M_1 cells descending from an ancestor that acquired the first oncogenic event at $t_1 \leq T$. Note that the number of M_1 cells evolves at rate $\lambda_2 - \delta_2/r$ during contraction. Finally, in Case III (both oncogenic events occur during precursor decay), $t_1 > T$ and $t_2 > T$ and accordingly we have

$$M_1(t_2|t_1; t_1 > T) = \mu_1\lambda_2 N(t_1)e^{(\lambda_2 - \delta_2/r)(t_2 - t_1)} \tag{S3}$$

M_1 cells at t_2 descending from an ancestor that acquired the first oncogenic event at t_1. Here, due to the selective advantage during decay, M_1 cells arising from earlier t_1 have more progeny than M_1 cells arising from later t_1. Integration yields the probability that the ECA dates back to latest t_1, given that the MRCA dates back to t_2 and $t_2 > T$:

$$P(t_1|t_2; t_2 > T) = \begin{cases} \dfrac{\int_0^{t_1} M_1(t_2|\tau; \tau \leq T)\, d\tau}{\int_0^{T} M_1(t_2|\tau; \tau \leq T)\, d\tau + \int_T^{t_2} M_1(t_2|\tau; \tau > T)\, d\tau}; \; t_1 \leq T, \\ \dfrac{\int_0^{T} M_1(t_2|\tau; \tau \leq T)\, d\tau + \int_T^{t_1} M_1(t_2|\tau; \tau > T)\, d\tau}{\int_0^{T} M_1(t_2|\tau; \tau \leq T)\, d\tau + \int_T^{t_2} M_1(t_2|\tau; \tau > T)\, d\tau}; \; t_1 > T. \end{cases}$$

Substituting equations (S2) and (S3) yields equation (16b).

5

Supplementary Note 2. Modeling neuroblastoma initiation in an initially expanding and then homeostatic population.

(a) Derivation of equations (14d, e). If which the initial phase of exponential expansion is followed by homeostatic turnover, proliferation and loss are balanced and thus $\lambda_2 = \delta_2$. Hence equation (8) becomes

$$N(t) = \begin{cases} e^{(\lambda_1-\delta_1)t}, & t \leq T, \lambda_1 > \delta_1 \\ e^{(\lambda_1-\delta_1)T}, & t > T. \end{cases}$$

Repeating the steps outlined in **Supplementary Note 1a** with this modification results in an equations (14d, e).

(b) Derivation of equations (17). In the case of progenitor homeostasis, the probabilities for acquiring the ECA at time t_1 are given by equations (17), as follows. Case I is unaffected by this modification. In Case II we have

$$M_1(t_2|t_1; t_1 \leq T) = \mu_1\lambda_1 N(T) e^{\lambda_2(1-1/r)(t_2-T)}$$

M_1 cells descending from an ancestor that acquired the first oncogenic event at t_1. As before, $M_1(t_2|t_1; t_1 \leq T)$ is independent of t_1. In Case III we have

$$M_1(t_2|t_1; t_1 > T) = \mu_1\lambda_2 N(T) e^{(\lambda_2(1-1/r))(t_2-t_1)}$$

M_1 cells descending from an ancestor that acquired the first oncogenic event at t_1. In this case, more progeny descends from earlier values of t_1 than from later values, as before. Together, the probability that the ECA dates back to latest t_1, given that the MRCA dates back to t_2 and $t_2 > T$ is then computed using equation (11) with the updated expressions for $M_1(t_2|t_1)$. This yields equation (17).

1. Iwasa, Y., Nowak, M.A. & Michor, F. Evolution of resistance during clonal expansion. *Genetics* **172**, 2557-2566 (2006).
2. Haeno, H., Iwasa, Y. & Michor, F. The evolution of two mutations during clonal expansion. *Genetics* **177**, 2209-2221 (2007).
