# Supplementary material for: Neuroblastoma arises in early fetal development and its evolutionary duration predicts outcome
Source: Nat Genet. 2023 Mar 27;55(4):619–30. doi: 10.1038/s41588-023-01332-y (PMC10101850; doi:10.1038/s41588-023-01332-y)
Supplement: Supplementary file 2 — Reporting summary. [file 41588_2023_1332_MOESM2_ESM.pdf]

nature portfolio

Corresponding author(s): Thomas Höfer, Frank Westermann

Last updated by author(s): Dec 15, 2022

Reporting Summary

Nature Portfolio wishes to improve the reproducibility of the work that we publish. This form provides structure for consistency and transparency in reporting. For further information on Nature Portfolio policies, see our Editorial Policies and the Editorial Policy Checklist.

Statistics

For all statistical analyses, confirm that the following items are present in the figure legend, table legend, main text, or Methods section.

n/a	Confirmed	
☐	☒	The exact sample size (*n*) for each experimental group/condition, given as a discrete number and unit of measurement
☒	☐	A statement on whether measurements were taken from distinct samples or whether the same sample was measured repeatedly
☐	☒	The statistical test(s) used AND whether they are one- or two-sided<br>*Only common tests should be described solely by name; describe more complex techniques in the Methods section.*
☐	☒	A description of all covariates tested
☐	☒	A description of any assumptions or corrections, such as tests of normality and adjustment for multiple comparisons
☐	☒	A full description of the statistical parameters including central tendency (e.g. means) or other basic estimates (e.g. regression coefficient) AND variation (e.g. standard deviation) or associated estimates of uncertainty (e.g. confidence intervals)
☐	☒	For null hypothesis testing, the test statistic (e.g. *F*, *t*, *r*) with confidence intervals, effect sizes, degrees of freedom and *P* value noted<br>*Give P values as exact values whenever suitable.*
☐	☒	For Bayesian analysis, information on the choice of priors and Markov chain Monte Carlo settings
☒	☐	For hierarchical and complex designs, identification of the appropriate level for tests and full reporting of outcomes
☐	☒	Estimates of effect sizes (e.g. Cohen's *d*, Pearson's *r*), indicating how they were calculated

Our web collection on statistics for biologists contains articles on many of the points above.

Software and code

Policy information about availability of computer code

Data collection: No software was used for data collection.

Data analysis: Analysis was performed with R (v3.6.0 and v4.0.0) and python v 3.6.1.

R packages:
openxlsx v4.1.5
ggsignif v0.6.0
ggbeeswarm v0.6.0
RColorBrewer v1.1-2
HDInterval v0.2.2
cdata v1.1.8
moments v0.14
Hmisc v4.4-0
scales v1.1.1
bedr v1.0.7
ggplot2 v3.3.2
ggbio v1.34.0
ggpubr v0.4.0
gridExtra v2.3
circlize v0.4.10
pammtools v0.2.2

ComplexHeatmap v2.5.1
BSgenome.Hsapiens.UCSC.hg19 v1.4.3
MASS v7.3-51.6
GenomicRanges v1.38.0
Bioonductor v3.15
reshape2 v1.4.4
mixtools v1.2.0
dplyr v1.0.0
survminer v0.4.8
survival v3.1-12
wesanderson v0.3.6
cowplot v1.1.1
mobster v1.0.0
CNAqc v1.0.0
mmsig v0.0.0.9000

python packages:
SigProfilerMatrixGenerator v1.1.26
SigProfilerExtractor v1.1.1
pyABC v0.9.13

Custom code is available on github (https://github.com/hoefer-lab).

For manuscripts utilizing custom algorithms or software that are central to the research but not yet described in published literature, software must be made available to editors and reviewers. We strongly encourage code deposition in a community repository (e.g. GitHub). See the Nature Portfolio guidelines for submitting code & software for further information.

Data

Policy information about availability of data

All manuscripts must include a data availability statement. This statement should provide the following information, where applicable:

- Accession codes, unique identifiers, or web links for publicly available datasets
- A description of any restrictions on data availability
- For clinical datasets or third party data, please ensure that the statement adheres to our policy

Subsets of the whole genome sequencing and RNA sequencing data were part of previously published studies24,26. Data of these studies is deposited at the European Genome-Phenome Archive (https://www.ebi.ac.uk/ega/) under accession numbers EGAS00001004349 and EGAS00001001308. Additional whole genome sequencing data generated for this study is available at the European Genome-phenome Archive under the accession numbers EGAS00001004990 and EGAS00001006533. In accordance with the laws of data protection, data is deposited under controlled access. Access can be granted by contacting Frank Westermann (f.westermann@kitz-heidelberg.de) and requires a data access agreement. Requests will be replied to within four weeks. Variant calls (SNVs, Indels, SVs and CNVs), mutational signatures, model fits and a summary on the mutation profile and the modeling results for each tumor can be accessed on Mendeley (http://dx.doi.org/10.17632/m9pwjbm7c8.1)70. All remaining data is available in the Supplementary information.

Human research participants

Policy information about studies involving human research participants and Sex and Gender in Research.

Reporting on sex and gender	Patients' sex is reported in Supplementary Table 1.
Population characteristics	See Supplementary Table 1 for age at diagnosis and survival information.
Recruitment	Cohorts of primary and relapsed neuroblastoma tumors were retrospectively analyzed. Tumor material was collected as part of the diagnostic workflow of the German Neuroblastoma trial by the Society for Pediatric Oncology and Hematology (GPOH) and collected in the Neuroblastoma tumor bank.
Ethics oversight	All trials were approved by the Ethics Committee of the Medical Faculty, University of Cologne and collection and use of all tumor tissue material was approved (NB97, NB2004, NB2016 registry). All patients or their parents signed an informed consent.

Note that full information on the approval of the study protocol must also be provided in the manuscript.

Field-specific reporting

Please select the one below that is the best fit for your research. If you are not sure, read the appropriate sections before making your selection.

☒ Life sciences ☐ Behavioural & social sciences ☐ Ecological, evolutionary & environmental sciences

For a reference copy of the document with all sections, see nature.com/documents/nr-reporting-summary-flat.pdf

Life sciences study design

All studies must disclose on these points even when the disclosure is negative.

Sample size	A unique cohort of ultra-deep WG sequenced neuroblastomas was analyzed, primarily limited by availability of appropriate samples. Available sample size allowed meaningful statistical analysis.
Data exclusions	No data were excluded from the analyses
Replication	Individual tumor samples were analyzed and compared by statistical analysis. The analysis was validated in an independent cohort.
Randomization	n/a. This is a retrospective analysis of tumor material that was collected as part of the diagnostic workflow of the German Neuroblastoma trial by the Society for Pediatric Oncology and Hematology (GPOH) and collected in the Neuroblastoma tumor bank. No case-control design was involved.
Blinding	n/a. This is a retrospective analysis of tumor material that was collected as part of the diagnostic workflow of the German Neuroblastoma trial by the Society for Pediatric Oncology and Hematology (GPOH) and collected in the Neuroblastoma tumor bank. No case-control design was involved.

Reporting for specific materials, systems and methods

We require information from authors about some types of materials, experimental systems and methods used in many studies. Here, indicate whether each material, system or method listed is relevant to your study. If you are not sure if a list item applies to your research, read the appropriate section before selecting a response.

Materials & experimental systems

n/a	Involved in the study	
☒	☐	Antibodies
☒	☐	Eukaryotic cell lines
☒	☐	Palaeontology and archaeology
☒	☐	Animals and other organisms
☐	☒	Clinical data
☒	☐	Dual use research of concern

Methods

n/a	Involved in the study	
☒	☐	ChIP-seq
☒	☐	Flow cytometry
☒	☐	MRI-based neuroimaging

Clinical data

Policy information about clinical studies

All manuscripts should comply with the ICMJE guidelines for publication of clinical research and a completed CONSORT checklist must be included with all submissions.

Clinical trial registration	This is a retrospective analysis of tumor material from patients being enrolled in the neuroblastoma trials of the GPOH and/or INFORM registry trial.
Study protocol	n/a. This is a retrospective analysis of tumor material from patients being enrolled in the neuroblastoma trials of the GPOH and/or INFORM registry trial.
Data collection	n/a. This is a retrospective analysis of tumor material from patients being enrolled in the neuroblastoma trials of the GPOH and/or INFORM registry trial.
Outcomes	n/a. This is a retrospective analysis of tumor material from patients being enrolled in the neuroblastoma trials of the GPOH and/or INFORM registry trial.
